# Ought Members of the Active Medical Staff to Sit upon Hospital Boards?

**Published:** 1890-03-15

**Authors:** Alexander Wallace

**Affiliations:** Physician. Colchester


### Editor's Letter-Box.

### OUGHT MEMBERS OF THE ACTIVE MEDICAL STAFF TO SIT UPON HOSPITAL BOARDS?

One of the greatest elements of friction in the management of a general hospital is the antagonism between the hospital committee or board, and the medical staff of the institution. The committee, not unfrequently, consider themselves as the masters of the situation, and the medical staff as their officers, and as such subject to their irony. The medical staff, mostly honorary, often men of eminence in their profession, consider their work as a matter of the highest importance to the hospital, and in its nature not to be understood by the committee, and, therefore, not to be found fault with by them. They consider themselves as appointed by and responsible to the governors of the hospital only for the due performance of their duties, and holding as responsible honorary officers, such a position as ought to free them from being heckled by the committee. Now such views, if held on either side, are likely sooner or later to damage any institution; on the one hand a feeling is apparent on the part of the committee that they do not want the interference of the medical staff, and on the other hand the staff feel they would do their work better if the committee would leave them alone. Would it not be better to avoid all this by looking at the professional work and the management of the hospital as two separate co-existing matters, by consolidating the medical staff, including the consulting staff, into a medical board, responsible for all the medical and surgical work of the hospital, and by making the chairman *pro tem.* of the medical board, and the senior consulting physician and senior consulting surgeon members of the committee? Thus the whole work of the staff of the hospital is revised by themselves, their minutes are open to the inspection of the committee, and they have the advantage of being represented if necessary on the committee, when their views collectively may be advanced for the consideration of the committee. On the other hand the committee, if they see occasion for blame, refer the matter to the medical board rather than address themselves to the individual. In this way friction is lessened. The medical board and weekly committee have each their separate departments to preside over, while the proportion of medical men on the weekly committee can never so preponderate as to do more than to present the collective opinion of the staff. Each body being supreme in their work is unlikely to criticise the other, and both recognizing the importance of the work of the other, are more inclined to co-operate with than retard each other. Such, since 1881, has been the constitution of the Colchester Hospital, and though differences of opinion have, as they always do, from time to time arisen, yet, on the whole, the system has worked well, and the efficiency of the hospital has largely increased.

Alexander Wallace, M.D., Physician.

Colchester, February 25, 1890.

